# Commentary: The impact of autoimmune diseases on delirium risk in critically ill patients: a propensity score matching multicenter analysis

**DOI:** 10.3389/fmed.2025.1692433

**Published:** 2025-11-13

**Authors:** Peishan Yu, Rui Zhang, Shaoyang Cui

**Affiliations:** Shenzhen Hospital (Futian) of Guangzhou University of Chinese Medicine, Shenzhen, Guangdong, China

**Keywords:** autoimmune diseases, delirium, critically ill patients, IL-17, TNF-α

## 1 Introduction

Importantly, positioning AID within the delirium framework also raises the possibility that systemic immune priming may not only alter acute vulnerability but also recalibrate long-term brain resilience. This perspective reframes delirium from a transient complication to a potential biomarker of accelerated neuro-immune aging in patients with autoimmune disease (AID). The provocative study by Huang et al. delivers a compelling message: AID independently amplify delirium risk in intensive care unit (ICU) patients (HR 2.376, *p* < 0.001). This multicenter analysis leverages robust methodology (PSM/IPTW) to illuminate a long-overlooked intersection between systemic autoimmunity and acute brain dysfunction (6). As neuroinflammation emerges as a cornerstone of delirium pathogenesis, this work forces us to confront a critical question: *Could AID represent the “missing link” explaining heterogeneity in delirium susceptibility beyond traditional risk factors?* Their findings challenge the prevailing clinical paradigm that primarily focuses on acute insults, urging a shift toward integrating chronic immune dysregulation into delirium risk models.

However, the observational nature of Huang et al.'s study necessitates cautious interpretation. The reliance on ICD codes for AID and delirium may introduce misclassification bias. Furthermore, while propensity score matching balances measured confounders, unmeasured variables—such as pre-existing cognitive impairment, ICU sedation practices, and detailed immunosuppressive regimens—could residually confound the observed relationship. Rather than undermining the study's validity, these limitations precisely these limitations that illuminate the path for future inquiry and frame the key unresolved dimensions we discuss below.

## 2 Key contributions and immediate clinical implications

Despite its limitations, the study by Huang et al. provides pivotal, large-scale evidence that should alter clinical consciousness in the ICU. By rigorously establishing AID as an independent risk factor for delirium, it mandates a shift in how we assess vulnerability. Clinicians must now recognize a patient's autoimmune status as a key component of delirium risk stratification, prompting heightened surveillance using tools like the CAM-ICU and ICDSC in this population. The dissociation between increased delirium incidence and unchanged mortality underscores that the burden of AID in the ICU is predominantly neurological, shifting clinical attention toward brain protection without the confounding fear of increasing mortality. This insight is immediately actionable, suggesting that for AID patients, resource allocation could be shifted toward enhanced neurological monitoring and early non-pharmacological interventions, such as sleep hygiene and early mobilization protocols.

## 3 Unresolved dimensions and future research imperatives

The findings by Huang et al. serve as a starting point, not a conclusion. They raise several critical questions that demand exploration.

### 3.1 Subtype heterogeneity and mechanistic specificity

**Figure 1:**
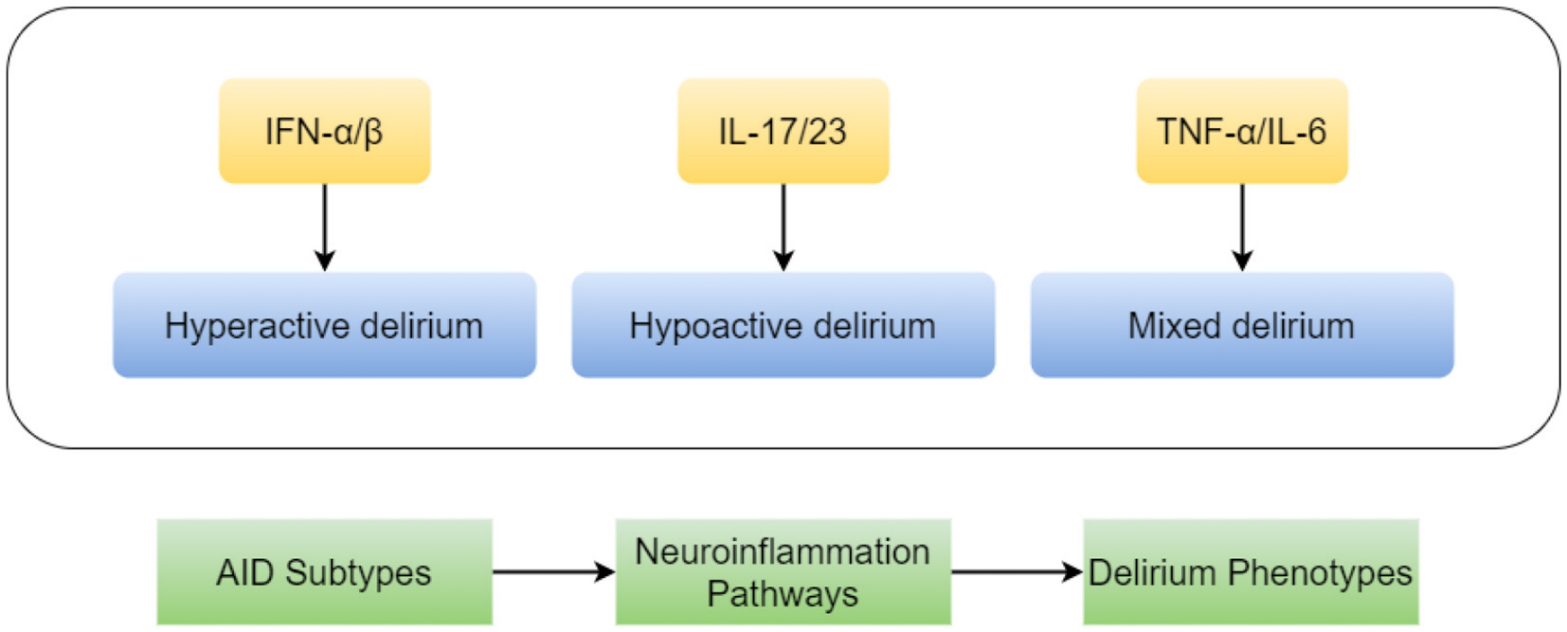
A hypothetical diagram illustrating the heterogeneous mechanisms by which autoimmune disease subtypes contribute to delirium. Distinct autoimmune diseases (e.g., Systemic Lupus Erythematosus, SLE, vs. Rheumatoid Arthritis, RA) drive specific neuroinflammatory responses by predominating different immune pathways (such as the type I interferon pathway vs. the IL-17 pathway). This heterogeneity may lead to diverse clinical phenotypes of delirium and influence responses to treatments (e.g., targeted biologic agents).

While the study confirms AID's aggregate risk effect, the silence on subtype stratification (e.g., SLE vs. rheumatoid arthritis) masks crucial biological diversity. Type I interferon-driven diseases (e.g., SLE) exhibit distinct neuroinflammation pathways (1) vs. IL-17-dominated pathologies (e.g., psoriasis) (2). These differences may translate to variable delirium clinical presentations (phenotypes) and therapeutic vulnerabilities. For instance, the pro-inflammatory cytokine IL-17, pivotal in psoriasis pathogenesis, has been directly implicated in blood-brain barrier disruption and neuroinflammation (2), suggesting a plausible mechanism for increased delirium risk in this AID subtype. Future studies must dissect subtype-specific mechanisms, as conceptually summarized in Figure 1.

Addressing this limitation requires hybrid study designs that merge registry-based cohorts with prospective pharmacovigilance platforms. Such integration would enable disentangling the differential neurocognitive impact of steroid pulses, chronic immunosuppressants, and next-generation biologics in real-world ICU populations.

However, biomarker-driven prediction must be embedded within longitudinal follow-up frameworks that incorporate serial cognitive testing and functional outcomes. Without such integration, the field risks reducing biomarkers to associative signals rather than actionable prognostic tools guiding rehabilitation or neuroprotective trials.

### 3.2 The double-edged sword of immunosuppression

While the study appropriately adjusts for steroid exposure as a covariate, it overlooks critical nuances in immunosuppressive therapy dynamics that may fundamentally alter delirium risk trajectories. The temporal pattern of steroid administration—particularly high-dose pulse therapy during acute autoimmune flares (e.g., lupus nephritis)—may paradoxically induce delirium through glutamatergic excitotoxicity and blood-brain barrier disruption, whereas chronic low-dose maintenance regimens could confer neuroprotection via sustained inflammation control. Furthermore, the impact of advanced biologics remains unaddressed; B-cell depletion agents such as rituximab may attenuate neuroautoantibody production and microglial activation (3), potentially reducing delirium incidence—a dimension inherently obscured by registry data's inability to capture targeted immunomodulation effects. This underscores the critical need to dissect the specific immune mechanisms at play, as studies have shown that distinct peripheral immune profiles, such as alterations in monocyte subsets and inflammatory cytokines, are significantly associated with delirium risk and mortality in critically ill populations (7). This limitation underscores how database studies may mask critical treatment-effect heterogeneity across the autoimmune disease spectrum.

Equally, preventive strategies such as early mobilization, sleep hygiene protocols, and delirium-preventive bundles must be re-examined in AID subgroups, as their efficacy may be modulated by baseline immune activation. Embedding AID-specific risk metrics into standard ICU delirium bundles could bridge the gap between discovery and practice.

### 3.3 Beyond acute episodes: long-term neurocognitive trajectories

The focus on in-ICU delirium (29.7% incidence) sidesteps a pivotal concern: *Do AID patients sustain accelerated cognitive decline post-ICU?* Emerging data suggest that the chronic immune activation in AID pre-activates the brain's immune cells (microglia), creating a vulnerable state akin to a “tinderbox.” This is particularly concerning given that post-intensive care cognitive impairment is highly prevalent, affecting nearly half of all survivors in the first 3-6 months and approximately 30% at one year (8). In this scenario, an acute insult like sepsis can act as a match, sparking a persistent and damaging neuroinflammatory response that outlasts the initial ICU stay. We propose integrating biomarkers of axonal injury, such as neurofilament light chain (NfL), which has been shown to predict long-term cognitive outcomes in critically ill patients (4).

## 4 Discussion

Huang et al. establish foundational evidence positioning autoimmune diseases (AID) as significant delirium risk amplifiers, yet this necessitates urgent translation into clinical action through three synergistic imperatives. First, developing precision risk stratification tools requires integrating AID-specific parameters such as disease activity metrics (e.g., SLEDAI for lupus), CNS-penetrating autoantibody profiles, and dynamic inflammatory trajectories like IL-6 kinetics to predict individual vulnerability. The ultimate goal is to move from a one-size-fits-all delirium bundle to a precision-guided, immune-informed prevention strategy. Second, targeted immunomodulation strategies must be prioritized—particularly trials of targeted biologics with potential CNS-sparing effects [e.g., anifrolumab, a type I interferon receptor antagonist approved for SLE (5)] in high-risk ICU cohorts—where the observed dissociation between elevated delirium incidence and unchanged mortality suggests neuroprotection can be achieved without compromising survival. The observed dissociation between elevated delirium incidence and unchanged mortality is pivotal here, as it suggests that neuroprotection can be pursued as a primary endpoint without compromising survival, thus de-risking the rationale for such clinical trials. Finally, research must extend beyond the ICU to understand long-term cognitive outcomes. Longitudinal studies are needed to dissect how autoimmunity increases the risk of post-ICU dementia. This work would directly test the “two-hit hypothesis”: the concept that the chronic inflammation of AID provides the first hit by sensitizing the brain, making it profoundly vulnerable to a second hit from the physiological stress of critical illness.

## 5 Conclusion

Huang et al. have successfully shifted the paradigm by positioning autoimmune diseases as a key modifier of delirium pathophysiology in the critically ill. The journey from this epidemiological association to improved patient outcomes, however, now begins. The imperative is to deconstruct the monolithic “AID” category into mechanistically distinct entities, to unravel the complex effects of immunomodulatory therapies, and to extend our vision beyond the ICU walls to the long-term cognitive health of survivors. By embracing these challenges, we can translate this foundational evidence into mechanism-guided interventions, ultimately pioneering targeted neuroprotection for this vulnerable population and illuminating the intricate dialogue between the immune system and the brain under duress.
